# The effect of mental health problems on having a ‘neither in employment nor in education or training’ period and the mediating role of high school dropout: a register-based study with a 14-year follow-up

**DOI:** 10.1136/jech-2024-222197

**Published:** 2025-03-26

**Authors:** Roos Hijdra, Joost Oude Groeniger, Alex Burdorf, Merel Schuring

**Affiliations:** 1 Department of Public Health, Erasmus MC, Rotterdam, Netherlands

**Keywords:** MENTAL HEALTH, EDUCATION, UNEMPLOYMENT

## Abstract

**Introduction:**

This study investigates (1) whether mental health problems among individuals aged 12–15 years impact (a) high school dropout (ages 16–20 years) and (b) having a ‘neither in employment nor in education or training’ (NEET) period (ages 21–26 years); (2) the process of mediation and interaction by high school dropout in the association between mental health problems and NEET; and (3) whether these associations differ based on (non-)employment and mental health problems of parents.

**Methods:**

Longitudinal register data were used (n=196 227). Log-linear regression analyses were used to assess the association between reimbursed medication for mental health problems and high school dropout or NEET period for at least 12 months. Causal mediation analysis was used to assess the mediation and interaction effects of high school dropout in the association between mental health problems and NEET. Stratified analyses were performed based on parental employment and mental health status.

**Results:**

Mental health problems were strongly associated with high school dropout (RR 1.96, 95% CI 1.88; 2.04) and NEET (RR 2.44, 95% CI 2.35; 2.52). High school dropout had a small mediating effect in the relationship between mental health problems and NEET. Individuals with parents with mental health problems or non-employment more often experienced high school dropout and being NEET, but the mediating effect of dropout on NEET was lower in these individuals.

**Conclusion:**

Preventing mental health problems early in the lifecourse is of paramount importance to promote educational outcomes and employment participation, but high school dropout only plays a marginal role in this relationship.

**WHAT IS ALREADY KNOWN ON THIS TOPIC**

**WHAT THIS STUDY ADDS**

- Mental health problems increased the likelihood of high school dropout and consequently becoming NEET, where high school dropout had a small mediating effect.
- Among individuals with parental non-employment and parental mental health problems, high school dropout and becoming NEET was more common, but with a lower mediating effect of dropout on NEET.

**HOW THIS STUDY MIGHT AFFECT RESEARCH, PRACTICE OR POLICY**

- These findings support the need for preventing mental health problems early in the life course to diminish the odds of becoming NEET.

## Introduction

 In 2016, 12% of European Union residents aged 15 to 24 years were neither in employment nor in education or training (NEET).^1^ This substantial group has poorer self-rated mental health compared with their peers and is more likely to develop substance use disorders and suicidal behaviour.^2 3^ Becoming NEET during early adulthood increases the risk of unemployment, dependence on governmental benefits, and poverty later in life.^4^ These are by themselves important predictors of worse living conditions and poorer health outcomes over the life course.^5^

There are various reasons why young people may become NEET, both voluntarily and involuntarily.^1^ One of the pathways through which childhood mental health may be an important determinant of becoming NEET is via high school dropout. Previous research revealed that almost one in four individuals who dropped out of high school experienced depressive symptoms during the prior 3 months.^6^ Additionally, dropping out of high school increases the likelihood of entering unemployment,^7^ as youth who leave school early are 12 times more likely to become NEET compared with youth who complete school.^8^ Furthermore, a Dutch study reported a mediating effect of high school dropout on the pathway from externalising mental health problems (eg, behavioural issues, anger, aggression) among men to becoming NEET, but not among women nor for internalising mental health problems.^9^

However, high school dropout may not only contribute to the relationship between mental health and NEET because high school dropout is more prevalent among children with mental health problems (ie, mediation)^9^ but also because there is a combined effect of mental health problems and high school dropout on NEET (ie, interaction). To inform policies and interventions, it is relevant to consider both of these processes and assess how they impact the relationship of mental health disorders with having a NEET period.^10^ Therefore, this study considers both the mediation and interaction effect of high school dropout in the relationship between mental health problems and having a NEET period.

Additionally, the family situation of young people may also play a role in the associations between mental health, high school dropout and NEET. A mental disorder may influence parenting responsibilities by not being able to provide the needed support.^11^ Contrarily, it may also be that parents who have more experience with mental health disorders themselves are better able to limit the negative impact of mental health disorders among their children.^12^ Yet, the little research which exists on how the employment status or mental health problems of parents influences the relationship between children’s mental health on NEET shows inconsistent results.^13^,^15^

Our study advances previous literature on NEET in interrelated ways. First, we use 14 years of follow-up register data covering the entire Dutch population compared with previous studies that only followed youth for a brief period (1–2 years),^16 17^ or used self-reported data with a smaller study population.^9 18^ Second, whereas extant research only focused on high school dropout or mental health,^18 19^ we investigate the inter-relationships between mental health disorders, high school dropout and NEET, and examine to what extent these relationships are dependent on parental characteristics. By doing so, this study addresses the following research questions:

1. What is the association of mental health problems during childhood with high school dropout during adolescence and NEET during the early years of adulthood?
2. What is the mediating and interaction effect of high school dropout during adolescence in the relationship between mental health problems during childhood and NEET during the early years of adulthood?
3. Are these associations influenced by employment participation and mental health problems of the parents?

## Methods

### Study design and population

A longitudinal study was conducted with register data from Statistics Netherlands with 14 years of follow-up (2006–2020). Statistics Netherlands provided information on all Dutch residents on prescribed medication for mental health problems, high school dropout, NEET outcomes, sociodemographic factors and parental information on employment and prescribed medication. Informed consent was not needed for this study because authorised research institutes are allowed by law to use pseudonymised register-based data for research purposes. The Medical Ethical Committee of Erasmus MC Rotterdam waived the requirement for formal ethical application as the Medical Research Involving Human Subjects Act does not apply to the current study (MEC-2023-0376).

**Figure 1:**
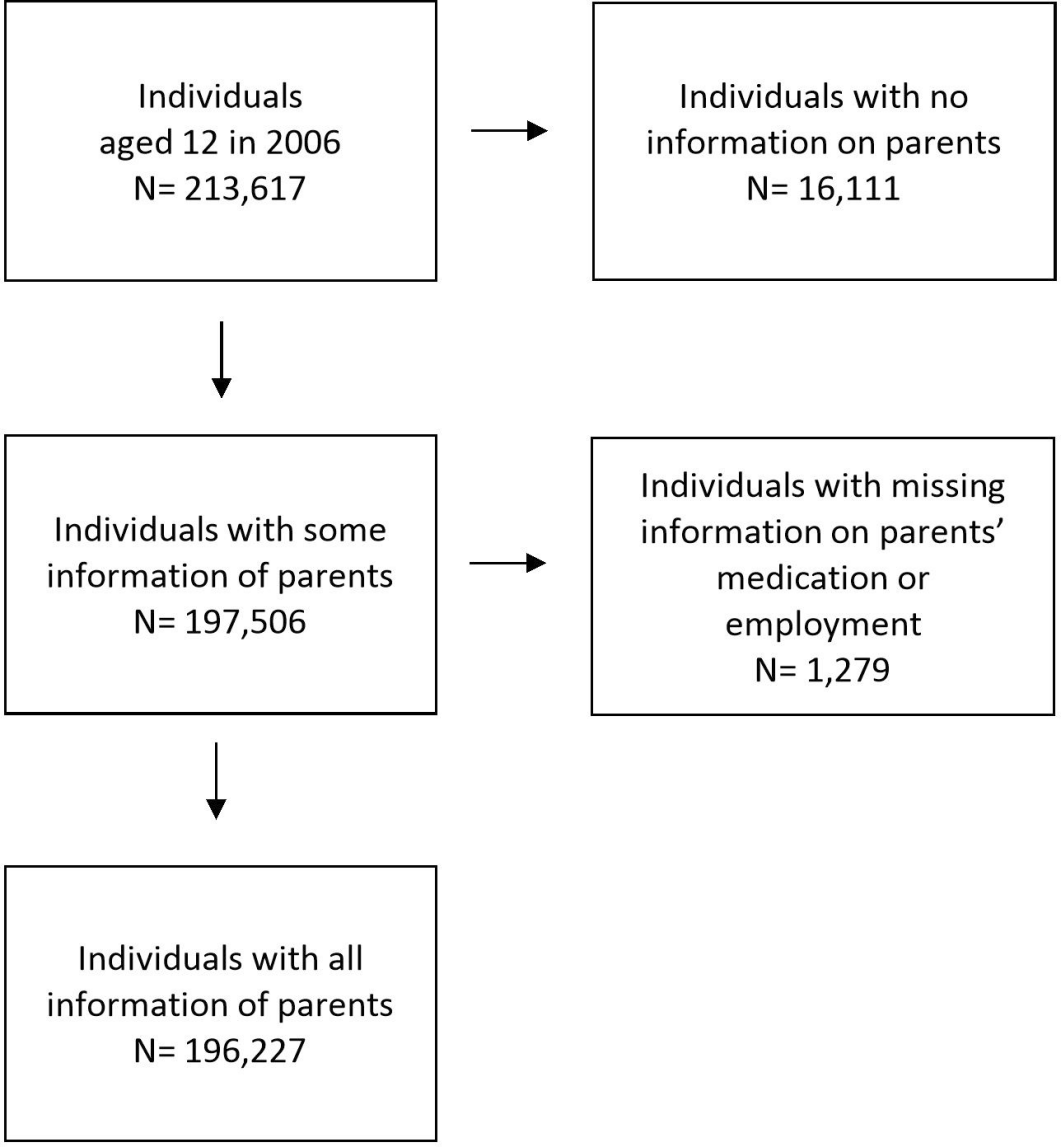
Flowchart of participant to display the inclusion and exclusion criteria.

Individuals were included if they were born in 1994 (n=2 13 617). Individuals were excluded because there was missing information on who the parents were (n=16 111) or on parental employment and prescribed medication (n=1279), resulting in a study population of 196 227 individuals (figure 1). Individuals without data of their parents actually had a lower prevalence of mental disorders and high school dropout, while the percentage of NEET was slightly higher (online supplemental table 1). In case an individual died or emigrated during the follow-up period, they were censored from the moment of their death or emigration onwards.

### Mental health problems

Statistics Netherlands provided information on purchased medication reimbursed by the health insurance per year between 2006 and 2009 (individuals aged 12 to 15). Based on WHO ATC codes, medication types for mental health problems were identified following the procedure described by Porru *et al*^20^ and Huber *et al*.^21^ The five categories included antipsychotics (for psychotic disorders, bipolar disorder or severe depression), anxiolytics (for anxiety or epilepsy), hypnotics and sedatives (for insomnia, anxiety or epilepsy), antidepressants (for depression, anxiety, post traumatic stress disorder or obsessive compulsive disorder) and psychostimulants (for attention deficit hyperactivity disorder).^20^ Individuals were classified as having a mental health problem if they were reimbursed medication for the selected mental health problem at least once between the ages of 12 and 15 years. The category ‘any mental health problem’ consists of individuals who were prescribed medication for any mental disorder at least once during the selected period.

### High school dropout

Yearly data on whether someone is currently following education or has dropped out of high school were obtained from Statistics Netherlands. These data were used from September of 2010 until July of 2014 (study population aged 16 up to 20 years). Having a high school dropout period was defined as not being registered at an educational institution for at least one school year. Second, it was dichotomised into having at least one high school dropout period or not having a high school dropout period during this time. After dropping out of high school, individuals can immediately enter employment or become NEET. It is also possible that individuals return to high school after their high school dropout period, but this could not be measured in the current study.

### NEET

Statistics Netherlands provided information on participation in education or employment for each month between January of 2015 and December of 2020 in which the study population was aged 21 up to 26 years. This information was used to identify months in NEET (no education or employment). In the current study, a NEET period was defined as being NEET for at least 12 consecutive months, in order to exclude individuals who dropped out and continued education in the next school year.

### Sociodemographic factors

Register data were obtained on age, sex and migration background in 2006. Migration background was based on the country of birth of the parents and was divided into five categories: Dutch, Moroccan, Turkish, Surinamese-Antillean, and other.

### Parents

Through household information of Statistics Netherlands, individuals were linked to others in the same household. Parents were identified by being born between 1950 and 1978, but the category may also include caregivers or siblings who are at least 16 years older. Parental characteristics were measured at baseline (2006) for a single year, because otherwise ‘future’ parental characteristics would have been included which were not present at the time of children’s mental health problems or high school dropout. Monthly information on employment status in 2006 was used to determine the parents’ employment status. An employed person was defined as being employed for at least 9 months in 2006. A dichotomous variable for parental employment was defined as at least one parent was employed. Likewise, a dichotomous variable for parental mental health problems was defined as at least one parent who had a mental health problem.

### Statistical analysis

First, descriptive statistics were used to describe the individual and household characteristics of the study population. Second, the prevalence of mental health problems, high school dropout and NEET was described for subgroups based on demographic and parental factors. Third, log-linear regression analysis was used to investigate the association of mental health problems with having a high school dropout period and having a NEET period, while adjusting for migration background and sex of the individual as well as parental mental health and employment status.

Causal mediation analysis was used to investigate the mediation and interaction effect of high school dropout on the relation between mental health problems and employment outcomes. To do so, we conducted a four-way decomposition analysis^22^ (on the excess relative risk scale) that decomposes the total effect of mental health problems on NEET into four components of mediation and interaction: (1) the controlled direct effect (CDE) is the effect that having mental health problems would have on becoming NEET, if nobody dropped out of high school; (2) the reference interaction (INTref) is the combined effect of having mental health problems and high school dropout on becoming NEET, if having a high school dropout period is not necessary for becoming NEET; (3) the mediated interaction (INTmed) is the combined effect that having mental health problems and having a high school dropout period on becoming NEET, if having a high school dropout period is necessary for becoming NEET; (4) the pure indirect effect (PIE) is the effect of having mental health problems on becoming NEET due to its effect on having a high school dropout period. Finally, this four-way decomposition analysis was stratified based on parental mental health problems and parental employment status. Descriptive statistics and log-linear regression analyses were performed in SPSS V.25 while the mediation analysis was performed in R V.4.2.3, with the CMAverse package.

## Results

1 in 15 individuals aged 12 years in 2006 in the Netherlands (6.7%) experienced mental health problems at least once between the ages of 12 and 15. A slightly higher proportion experienced at least one high school dropout period between the ages of 16 and 20 (10.6%). Between the ages of 21 and 26, one out of eight individuals experienced a NEET period of at least 12 consecutive months (12.8%). Individuals with at least one parent with mental health problems had a higher prevalence of (1) mental health problems themselves (9.7%), (2) a high school dropout period (13.8%) and (3) a NEET period (16.9%). Individuals with non-employed parents had a higher prevalence of high school dropout (18.2%) and NEET (27.3%) (table 1).

**Table 1:** Individual characteristics and the prevalence of mental health problems (12–15 years), high school dropout (16–20 years) and having a NEET period of at least 1 year (21–26 years) among Dutch individuals born in 1994

	Total N (%)	Any mental health problem N (%)	Having a high school dropout period N (%)	Having a NEET period N (%)
Total	196 227	13 138 (6.7)	20 879 (10.6)	25 087 (12.8)
Sex				
Female	96 021 (48.9)	4040 (4.2)	8661 (9.0)	12 017 (12.5)
Male	100 206 (51.1)	9098 (9.1)	12 218 (12.2)	13 070 (13.0)
Migration background				
Native Dutch	154 251 (78.6)	11 206 (7.3)	14 630 (9.5)	16 375 (10.6)
Moroccan	6132 (3.1)	241 (3.9)	1117 (18.2)	1959 (31.9)
Turkish	7055 (3.6)	234 (3.3)	1230 (17.4)	1608 (22.8)
Surinamese/Antillean	7087 (3.6)	343 (4.8)	1139 (16.1)	1564 (22.1)
Other	21 702 (11.1)	1114 (5.1)	2763 (12.7)	3581 (16.5)
Any mental health problem
Yes No	13 138 (6.7) 183 089 (93.3)		2707 (20.6) 18 172 (9.9)	3704 (28.2) 21 383 (11.7)
Parent with mental health problems				
No parent	148 549 (75.7)	8512 (5.7)	14 316 (9.6)	17 025 (11.5)
At least one parent	47 678 (24.3)	4626 (9.7)	6563 (13.8)	8062 (16.9)
Employed parent				
At least one parent	185 726 (94.6)	12 522 (6.7)	18 973 (10.2)	22 222 (12.0)
No parent	10 501 (5.4)	616 (5.9)	1906 (18.2)	2865 (27.3)

Table 2 shows that having any mental health problem had a strong estimated effect on both high school dropout (RR 1.96, 95% CI 1.88; 2.04) and becoming NEET (RR 2.44, 95% CI 2.35; 2.52). All medication categories showed substantial estimated effects on having a high school dropout period and becoming NEET, with only minor differences between the different medication categories. The strongest estimated effect among all medication categories on high school dropout was found for antidepressants (RR 2.23, 95% CI 1.99; 2.50), while anxiolytics had the weakest estimated effect (RR 1.41, 95% CI 1.27; 1.55). The strongest estimated effect on having a NEET period was found for antipsychotics (RR 3.92, 95% CI 3.69; 4.17), which was followed by hypnotics and sedatives (RR 2.69, 95% CI 2.33; 3.11).

**Table 2:** Estimated effects of mental health problems (12–15 years) on high school dropout (16–20 years) and having a NEET period of at least 1 year (21–26 years) (N=196 227)

	Total N (%)	High school dropout RR (95% CI)	NEET RR (95% CI)
Any mental health problem	13 138 (6.7)	1.96 (1.88; 2.04)	2.44 (2.35; 2.52)
Psychostimulants	8746 (4.5)	2.01 (1.92; 2.11)	2.11 (2.02; 2.21)
Anxiolytics	2596 (1.3)	1.41 (1.27; 1.55)	2.41 (2.25; 2.59)
Antipsychotics	2178 (1.1)	2.01 (1.84; 2.19)	3.92 (3.69; 4.17)
Hypnotics and sedatives	522 (0.3)	1.82 (1.49; 2.21)	2.69 (2.33; 3.11)
Antidepressants	1262 (0.6)	2.23 (1.99; 2.50)	2.68 (2.44; 2.95)

Adjusted for migration background and sex of the individuals and employment status and mental health status of their parents.

In table 3, a small estimated mediation effect (PIE) (ERR 0.19, 95% CI 0.17; 0.20) of high school dropout was shown between having any mental health problem on having a NEET period. This estimated effect was similar among the various categories of medication for mental health problems. However, the negative INTref (ERR −0.02, 95% CI −0.04; 0.00) and the negative INTmed (ERR −0.02, 95% CI −0.04; 0.00) imply that the combined estimated effect of high school dropout and mental health problems on having a NEET period was slightly smaller than the product of their estimated individual effect. Lastly, table 3 shows that if nobody dropped out of high school, the excess relative risk of becoming NEET with mental health problems would be 1.33 (CDE) (95% CI 1.24; 1.42). Although the INTref and INTmed are similar among the different medication categories, large differences are set out in the CDE, ranging from an ERR of 0.97 (95% CI 0.87; 1.07) among psychostimulants up to ERR 3.04 (95% CI 2.76; 3.31) for using antipsychotics.

**Table 3:** Four-way decomposition of the estimated effect of mental health problems (12–15 years) on having a NEET period (21–26 years), mediated by high school dropout (16–20 years) (N=196 227)

	High school dropout on NEET				
	Total effect ERR (95% CI)	CDE ERR (95% CI)	INTref ERR (95% CI)	INTmed ERR (95% CI)	PIE ERR (95% CI)
Any mental health problem	1.48 (1.39; 1.57)	1.33 (1.24; 1.42)	−0.02 (−0.04; 0.00)	−0.02 (−0.04; 0.00)	0.19 (0.17; 0.20)
Psychostimulants	1.14 (1.05; 1.24)	0.97 (0.87; 1.07)	−0.01 (−0.04; 0.01)	−0.01 (−0.04; 0.02)	0.20 (0.18; 0.21)
Anxiolytics	1.46 (1.29; 1.64)	1.47 (1.29; 1.65)	−0.07 (−0.12; −0.01)	−0.03 (−0.05; 0.00)	0.08 (0.05; 0.11)
Antipsychotics	3.06 (2.81; 3.31)	3.04 (2.76; 3.31)	−0.09 (−0.15; −0.03)	−0.10 (−0.16; −0.03)	0.20 (0.17; 0.24)
Hypnotics and sedatives	1.72 (1.34; 2.15)	1.48 (1.07; 1.89)	0.04 (−0.08; 0.16)	0.03 (−0.07; 0.14)	0.16 (0.10; 0.22)
Antidepressants	1.72 (1.47; 2.00)	1.56 (1.28; 1.83)	−0.03 (−0.10; 0.04)	−0.04 (0.13; 0.05)	0.24 (0.20; 0.29)

Adjusted for migration background and sex of the individuals and employment status and mental health status of their parents

CDE, controlled direct effect; INTref, reference interaction; INTmed, mediated interaction; PIE, pure indirect effect

Table 4 sets out the results from the four-way decomposition stratified on parental employment status and mental health problems. This analysis reveals that among individuals with at least one parent with mental health problems, the estimated mediation effect was slightly smaller (PIE 0.16, 95% CI 0.13; 0.18) compared with individuals with parents who did not use medication (PIE 0.21, 95% CI 0.19; 0.23). This smaller mediation effect was also observed among individuals whose parents were not in paid employment (PIE 0.07, 95% CI 0.03; 0.11), compared with individuals with at least one parent in paid employment (PIE 0.20, 95% CI 0.18; 0.22). Contrarily, among individuals with parents with mental health problems or who were non-employed, the presence of mental health problems during childhood had a smaller estimated controlled direct effect on becoming NEET during early adulthood compared with individuals with parents who did not have mental health problems or were employed. Finally, the negative estimates for the INTref and INTmed observed in the full sample were shown to be driven by individuals with non-employed parents or parents with mental health problems.

**Table 4:** Four-way decomposition of the estimated effect of mental health problems (12–15 years) on having a NEET period (21–26 years), mediated by high school dropout (16–20 years) and stratified on parental mental health and parental employment (N=196 227)

	High school dropout on NEET				
	Total effect ERR (95% CI)	CDE ERR (95% CI)	INTref ERR (95% CI)	INTmed ERR (95% CI)	PIE ERR (95% CI)
Individuals with at least one parent having mental health problems (N=47 678)	1.17 (1.05; 1.30)	1.10 (0.97; 1.23)	−0.05 (−0.09; −0.10)	−0.04 (−0.07; −0.01)	0.16 (0.13; 0.18)
Individuals with no parent having mental health problems (N=148 549)	1.65 (1.54; 1.78)	1.44 (1.32; 1.56)	0.00 (−0.03; 0.03)	0.00 (−0.03; 0.04)	0.21 (0.19; 0.23)
Individuals with no parent in employment (N=10 501)	0.70 (0.50; 0.93)	0.79 (0.56; 1.03)	−0.12 (−0.20; −0.03)	−0.04 (−0.08; 0.00)	0.07 (0.03; 0.11)
Individuals with at least one parent in employment (N=185 726)	1.54 (1.44; 1.63)	1.36 (1.27; 1.46)	−0.01 (−0.04; 0.01)	−0.01 (−0.04; 0.01)	0.20 (0.18; 0.22)

Adjusted for migration background and sex of the individuals and employment status and mental health status of their parents.

CDE, controlled direct effect; INTref, reference interaction; INTmed, mediated interaction; PIE, pure indirect effect

## Discussion

This study demonstrated that taking medication for mental health problems during childhood increased the likelihood of high school dropout during adolescence and NEET in early adulthood. High school dropout had a small mediating effect in the relationship between mental health problems and NEET. However, the effect of high school dropout on having a NEET period was slightly larger among individuals who did not take medication for mental health problems. This mediating effect remained small on comparing different parental characteristics.

In the current study, only a small mediating effect of high school dropout was found for all included types of medication for mental health problems on NEET. This indicates that the estimated association between mental health problems and NEET is strong, but high school dropout only marginally contributes to this association (between 5% and 18% depending on the type of medication). Another Dutch study partly supports this finding, where they found only a small mediating effect for having a high school diploma among men with externalising mental health problems (eg, behavioural issues, anger) on becoming NEET. Contrarily, among individuals with internalising mental health problems, no mediating effect was found.^9^ The absence of stronger mediation effects may be because individuals with mental health problems who drop out may return back to high school after this period and attain a degree. In the Netherlands, there is a compulsory school attendance law. This provides an incentive to return to high school after a dropout period and attain a high school diploma.^23^

As shown in the current study, individuals with precarious family circumstances (no parent in paid employment or at least one parent with mental health problems) have high school dropout and NEET rates that are up to twice as high compared with individuals with no precarious family circumstances. This is higher than reported in previous research.^24^,^27^ The results also suggest that the presence of mental health problems during childhood has a weaker direct effect on becoming NEET during early adulthood among individuals who grew up with precarious family circumstances compared with individuals who grew up without these circumstances. One explanation may be that for individuals with less precarious family circumstances, the relative importance of their own mental health problems may become stronger. Additionally, parents without mental health problems themselves might be less familiar with finding the right (way to) mental healthcare.^12^ This is also supported by the observed negative interaction between mental health problems and high school dropout, which only occurred among individuals who grew up with precarious family circumstances. This negative interaction may be due to individuals with mental health problems, or their parents, already being in a professional support system. This may enable them to limit the negative consequences of high school dropout on NEET.^12 28^

One of the strengths of this study is that it uses register-based data, capturing the entire population of the Netherlands that fits the inclusion criteria. Second, the four-way decomposition is a novel method to fully capture to what extent high school dropout contributes to the relationship between mental health problems and having a NEET period. However, this study also had limitations. Using data on medication for mental health problems means that only pharmacological treatment was identified. Information on medication prescription was available yearly and did not provide information on the amount of medication prescribed or used. The severity of the disorders was thus not accounted for. Furthermore, it was not possible to measure whether an individual participated in an employment programme from the municipality while they were non-employed. This means that those individuals were classified as being NEET. Finally, because we used longitudinal observational data, it is possible that the results of the analyses were influenced by unobserved confounders.

In conclusion, this study found that mental health problems in childhood are strongly associated with more NEET cases in early adulthood, but high school dropout only marginally contributed to this association. This suggests that it is of paramount importance to prevent, detect and treat mental health problems from an early age to promote employment and educational outcomes later in life, while focusing interventions and policies solely on reducing high school dropout among children with mental health problems will not be enough to tackle this societal challenge. Future studies are needed to better understand which mechanisms contribute to the negative impact of mental health problems on NEET, and how policies and interventions can effectively address these mechanisms.

## Supplementary material

10.1136/jech-2024-222197 online supplemental file 1

## Data Availability

Data may be obtained from a third party and are not publicly available.
