# Supplementary material for: The effect of mental health problems on having a ‘neither in employment nor in education or training’ period and the mediating role of high school dropout: a register-based study with a 14-year follow-up
Source: J Epidemiol Community Health. 2025 Mar 26;79(8):e222197. doi: 10.1136/jech-2024-222197 (PMC12322390; doi:10.1136/jech-2024-222197)
Supplement: online supplemental file 1 [file jech-79-8-s001.pdf]

Supplementary table 1 Prevalence of mental disorders, high school dropout and NEET among individuals with or without data of parents

	Individuals with data of parents (n=196,227)	Individuals without data of parents (n=17,390)
Mental disorders	13,138 (6.7)	510 (2.9)
High school drop out	20,879 (10.6)	1,510 (8.7)
NEET	25,087 (12.8)	2,475 (14.2)
